# Associations Between the Prevalence of Metabolic Syndrome and Sleep Parameters Vary by Age

**DOI:** 10.3389/fendo.2018.00234

**Published:** 2018-05-11

**Authors:** Olga E. Titova, Eva Lindberg, Sölve Elmståhl, Lars Lind, Helgi B. Schiöth, Christian Benedict

**Affiliations:** ^1^Department of Neuroscience, Uppsala University, Uppsala, Sweden; ^2^Department of Medical Sciences, Respiratory, Allergy and Sleep Research, Uppsala University, Uppsala, Sweden; ^3^Division of Geriatric Medicine, Department of Health Sciences, Clinical Research Centre (CRC), Lund University, Skåne University Hospital, Malmö, Sweden; ^4^Cardiovascular Epidemiology, Department of Medical Sciences, Uppsala University, Uppsala, Sweden

**Keywords:** sleep duration, sleep disturbance, sleep-disordered breathing, metabolic syndrome, age

## Abstract

**Objective:**

To examine whether the relationship between the metabolic syndrome (MetS) and various sleep parameters [sleep duration, symptoms of sleep-disordered breathing (SDB), and sleep disturbances] varies by age.

**Methods:**

Waist circumference, blood pressure, triglycerides, high-density lipoprotein cholesterol, and fasting glucose were used to determine MetS status in a cohort (*N* = 19,691) of middle-aged (aged 45–64 years) and older (aged ≥65 years) subjects. Habitual sleep duration (short, ≤6 h/day; normal, 7–8 h/day; and long ≥9 h/day), sleep disturbances (such as problems with falling and staying asleep), and symptoms of sleep-disordered breathing (SDB, such as snoring and sleep apneas) were measured by questionnaires.

**Results:**

Among the participants, 4,941 subjects (25.1%) fulfilled the criteria for MetS. In the entire sample, both short and long sleep durations were associated with higher prevalence of MetS as compared to normal sleep duration. When stratified by age, a similar pattern was observed for middle-aged subjects (<65 years old; prevalence ratio (PR) [95% CI], 1.13 [1.06–1.22] for short sleep and 1.26 [1.06–1.50] for long sleep duration). In contrast, in older individuals (≥65 years old), only long sleep duration was linked to a higher prevalence of MetS (1.26 [1.12–1.42]; *P* < 0.01 for sleep duration × age). In the entire cohort, having at least one SDB symptom ≥4 times per week was linked to an increased prevalence of MetS; however, the PR was higher in middle-aged subjects compared with older subjects (1.50 [1.38–1.63] vs. 1.36 [1.26–1.47], respectively; *P* < 0.001 for SDB × age). Finally, independent of subjects’ age, reports of sleep disturbances (i.e., at least one symptom ≥4 times per week) were associated with a higher likelihood of having MetS (1.12 [1.06–1.18]; *P* > 0.05 for sleep disturbance × age).

**Conclusion:**

Our results suggest that age may modify the associations between some sleep parameters and the prevalence of MetS.

## Introduction

The metabolic syndrome (MetS) is defined as a cluster of several cardio-metabolic risk factors. This includes hypertension, hyperlipidemia, hyperglycemia, reduced blood concentrations of high-density lipoprotein (HDL) cholesterol, and abdominal obesity (1). Accumulating evidence suggests that chronic poor sleep patterns can increase the risk of having MetS, or some of its components (2–4). For instance, in a recent meta-analysis involving 76,027 participants, short (defined as ≤6 h/day) and long sleep durations (defined as >8 h/day) were associated with increased risk of MetS (+27 and +23%, respectively), as compared with normal sleep duration (5). Additionally, suffering from sleep disturbances or sleep-disordered breathing (SDB) might also increase the risk of having MetS, or some of its components (6–10). This is for instance supported by findings of a recent meta-analysis demonstrating that subjects with obstructive sleep apnea (OSA; hallmarked by recurrent episodes of either partial or full cessation of breathing while asleep) were at 1.72 times higher risk for MetS (11), than those without OSA. Collectively, existing evidence indicates worrisome connections between poor sleep patterns and MetS.

Noteworthy, associations between poor sleep patterns and parameters of the MetS appear to vary by age. For instance, an association between short sleep duration (≤5 and 6 h) and prediabetes—defined by the authors as blood levels of glycated hemoglobin ≥5.7% (HbA1c; a proxy of the 3-month average plasma glucose concentration)—was found in Japanese subjects aged <40 years (*n* = 32,929). In contrast, long sleep duration (≥ 8 h) was associated with lower odds of having prediabetes in adults aged <40 years (12). In the same study, in older subjects (≥40 years; *n* = 42,543), short sleep duration (≤5 h) was associated with higher odds for prediabetes. Long sleep duration was, however, unrelated to prediabetes in older subjects (12). In a separate Korean study involving 5,393 subjects, it was found that young and middle-aged adults (19–64 years) who slept <6 h a day, compared to those who slept 7 h a day, had increased odds of hypertension. This association was not found among those aged ≥65 years (13). This is in line with a Spanish study (*N* = 3,686) in which self-reported sleep duration was not associated with hypertension among those aged 60 years and above (14). Overall, these results suggest that associations between poor sleep and components of the MetS may vary by age. However, the effects of age on the association between sleep parameters and the prevalence of MetS are not fully disentangled.

With this in mind, this study sought to examine whether relationships of sleep duration outside the recommended range (7–8 h per day), sleep disturbances (such as problems with falling and staying asleep), and SDB symptoms (such as snoring and sleep apneas) with MetS vary by age (45–64 vs. ≥65 years, common retirement age in Sweden). We hypothesized that poor sleep patterns increase the prevalence ratio (PR) of MetS.

## Materials and Methods

### Study Population

Our analysis was based on data from the EpiHealth cohort study (www.epihealth.se). At the time of retrieving data for the present analysis, the EpiHealth study was still recruiting subjects. This explains the difference in initial sample sizes between previous publications (15) and the present analysis. A detailed description of the study has been reported previously (16).

From the initial sample size (*n* = 20,534), two participants were excluded because their age was more than two SD apart from the population mean; 360 individuals were then excluded because of missing data on biochemical parameters or waist circumference. Finally, 437 participants had missing data on covariates, and 44 subjects provided no reports on sleep duration. After exclusions, data from 19,691 subjects (96% of the initial sample size) were available to investigate the association between sleep duration and MetS. From them, 19,142 (93% of the initial sample size) participants had complete data on sleep disturbance and 16,467 (80% of the initial sample size) on SDB symptoms.

The Ethics Committee at Uppsala University approved the general procedures of the EpiHealth study. All subjects gave written informed consent in accordance with the Declaration of Helsinki. An additional ethical approval for the current data analyses was obtained from the Ethics Committee at Uppsala University. A short description of the research proposal was displayed on EpiHealth’s homepage for 1 month which allowed participants in EpiHealth study to withdraw their consent.

### Metabolic Syndrome

Participants visited a test center for collection of physical measurements and blood samples (located in Malmö or Uppsala, Sweden). Blood pressure was recorded twice in the sitting position by trained personnel with automatic device (Omron, Kyoto, Japan). Waist circumference was measured at the umbilical level. Blood was collected for determination of fasting glucose, LDL- and HDL-cholesterol, and serum triglycerides at the hospital laboratory using an Architect Ci8200 analyzer (Abbott Laboratories, Abbott Park, IL, USA) (16). The same equipment as well as the same biochemical laboratory for analysis of glucose and lipids was used in both test centers (16).

Metabolic syndrome was defined as the presence of at least three of the following conditions: elevated waist circumference (≥102 cm for men; ≥88 cm for women); hypertriglyceridemia, defined as a serum triglyceride concentration ≥150 mg/dL [≥1.7 mmol/L]; low HDL cholesterol (<40 mg/dL [<1.0 mmol/L] for men and <50 mg/dL [<1.3 mmol/L] for women); elevated blood pressure (systolic ≥130 and/or ≥85 diastolic mmHg) or antihypertensive drug treatment (1); and elevated fasting glucose (≥110 mg/dL [≥6.1 mmol/L]) or drug treatment for diabetes (17).

### Sleep Variables

Participants were asked to indicate how many hour per day they usually sleep (“4 h or less,” “5h,” “6h,” “7h,” “8h,” “9h,” “10 h or more,” and “don’t know/don’t want to answer”). The answer “don’t know/don’t want to answer” was treated as missing value. Short sleep duration was defined as sleep ≤6 h per day, normal sleep duration corresponded to 7–8 h sleep per day, and sleep ≥9 h per day was defined as long sleep duration.

Sleep disturbances were determined based on the following symptoms: difficulties in falling asleep, early awakenings, difficulties getting back to sleep after nighttime awakenings, and disturbed sleep. Symptoms of SDB included witnessed sleep apnea and heavy snoring (witnessed or according to participate him/herself). Participants were required to indicate the frequency of each symptom by the following options: “never/seldom,” “1 to 3 times a month,” “1 to 3 times a week,” “4 or more times a week,” and “ don’t know/don’t want to answer.” Participants who reported that they suffered from at least one of the above-mentioned sleep disturbance symptoms for “4 or more times a week” were defined to have a sleep disturbance. Participants who indicated to experience either witnessed sleep apnea or heavy snoring for “4 or more times a week” (or both) was defined to have SDB symptoms. The option “don’t know/don’t want to answer” was treated as missing value.

### Covariates

Age and gender were recorded in the test center. Participants’ educational attainment, physical activity (PA) during leisure time, alcohol consumption frequency, and current smoking were assessed by the Internet-based questionnaire. Participants’ educational attainment was defined as primary and elementary school (up to 9 years of formal schooling), upper secondary school (up to 12 years of formal schooling), university, or other (e.g., further training). PA during leisure time was measured on an eight-point scale. A low level of PA was defined as spending most leisure time mostly sedentary or having light PA about 2–4 h per week, such as walking, gardening, and light housework, etc. A medium level of PA was defined as moderate PA at least 1–2 times a week, such as jogging, swimming, heavy gardening, etc., or light PA for more than 4 h per week or taking care of all the housework, both light and heavier. A high PA level was defined as more strenuous PA at least three times a week, such as playing tennis, swimming, and running, etc. Based on responses to six questions about smoking habits, the participants were assigned to non-smokers or smokers. Alcohol consumption frequency during the last 12 months was categorized as “never,” “≤1 time/week,” “2–3 times/week,” and “≥4 times/week,” yielding a four-level ordinal variable.

### Statistical Analysis

All statistical analyses were performed using SPSS version 22.0 (SPSS Inc., Chicago, IL, USA). Descriptive data are presented as mean (SD) for continuous variables and as percentages for categorical variables. The Pearson chi-square test was used to analyze group differences for categorical variables. Numerical data were analyzed with Mann–Whitney *U* test.

Binomial regression with log link function was performed to examine associations between the prevalence of MetS and sleep parameters. This statistical approach was used because the outcome (MetS) was common (25%) in the present cohort. Under such circumstances, log-binomial regression analysis is generally considered to be more conservative than logistic regression (18). PRs derived from log-binomial regression were adjusted for participants’ age (expressed in years), gender, educational attainment, leisure PA level, current smoking status, and alcohol consumption frequency.

Note that job-related questions (including those on shift work history) were presented only to participants who were still working at the time of the online survey (15). Hence, the shift work variable was not included in the main analysis due to a large proportion of missing values (*n* = 9,061, 44%) and risk of potential misclassification. Additionally, shift work was the only variable that was not significantly associated with MetS in fully adjusted models. Finally, comparison of regression models using Akaike’s Information Criterion (AIC) revealed that models not adjusted for shift work had lower AIC, indicating a better model fit.

Potential confounders were selected based on existing information on risk factors for impaired sleep and MetS using the method of directed acyclic graphs (DAGs) (19). DAGs is a widely used method to depict graphically assumed causal relationships between predictor, outcome, and confounder variables. Overall, no interactions between gender and sleep parameters were found (*P* > 0.05). Possible multiplicative interaction effects of sleep parameters with age (in y) on MetS risk were investigated in fully adjusted models (i.e., sleep parameter × age). We considered interaction present at *P* < 0.05.

The proportion of missing data on main exposure variables was less than 1% for sleep duration, 3% for sleep disturbance, 17% for SDB symptoms, and ≤1% for covariates. To assess if exclusions of subjects because of missing values could have affected the observed associations between sleep variables and the prevalence of MetS, we performed multiple imputation with the assumption that data were missing at random. The imputation procedure resulted in five imputation data sets.

## Results

### Study Population

Of the total 19,691 participants with complete data on sleep duration, 21% of middle-aged subjects (age <65 years) and 31.2% of older individuals (≥65 years) met criteria for MetS, respectively. Compared with middle-aged subjects, older individuals had more often an elevated waist circumference (≥102 cm for men; ≥88 cm for women), hypertension (systolic ≥130 and/or ≥85 diastolic mmHg or antihypertensive drug treatment), elevated serum triglycerides (≥150 mg/dL [≥1.7 mmol/L]), and elevated fasting glucose (≥110 mg/dL [≥6.1 mmol/L] or drug treatment for diabetes). In contrast, low HDL cholesterol level (<40 mg/dL [<1.0 mmol/L] for men and <50 mg/dL [<1.3 mmol/L] for women) did not differ between age groups. For more details, see Table 1.

**Table 1:** Participants’ characteristics, stratified by age.

		Age	
		
Parameter	Total	<65 years	≥65 years
Total participants, *n* (%)	19,691	11,804 (74.9)	7,887 (25.1)
Age, years, mean (SD)*	60.8 (8.5)	55.1 (5.7)	69.3 (3.1)
Metabolic syndrome, *n* (%)*	4,941 (25.1)	2,479 (21.0)	2,462 (31.2)
Females, *n* (%)*	11,139 (56.6)	7,078 (60.0)	4,061 (51.5)
Educational status, *n* (%)*			
*Primary/elementary school*	3,105 (15.8)	1,066 (9.0)	2,039 (25.9)
*Upper secondary school*	5,005 (25.4)	3,645 (30.9)	1,360 (17.2)
*University*	9,351 (47.5)	6,067 (51.4)	3,284 (41.6)
*Other*	2,230 (11.3)	1,026 (8.7)	1,204 (15.3)
Leisure PA level, *n* (%)*			
*Low*	7,834 (39.8)	4,520 (38.3)	3,314 (42.0)
*Medium*	8,258 (41.9)	4,675 (39.6)	3,583 (45.4)
*High*	3,599 (18.3)	2,609 (22.1)	990 (12.6)
Current smoking, *n* (%)*	1,493 (7.6)	989 (8.4)	504 (6.4)
Alcohol consumption frequency, *n* (%)*			
*Never*	1,110 (5.6)	646 (5.5)	464 (5.9)
*≤1 time/week*	10,484 (53.2)	6,597 (55.9)	3,887 (49.3)
*2–3 times/week*	6,316 (32.1)	3,810 (32.3)	2,506 (31.8)
*≥4 times/week*	1,781 (9.0)	751 (6.4)	1,030 (13.1)
Elevated waist circumference*			
*(≥102 cm for men; ≥88 cm for women)*	7,977 (40.5)	4,407 (37.3)	3,570 (45.3)
Elevated serum triglycerides*			
*(≥150 mg/dL [≥1.7 mmol/L])*	3,177 (16.1)	1,847 (15.6)	1,330 (16.9)
**Low high-density lipoprotein cholesterol level**			
*(<40 mg/dL [<1.0 mmol/L] for men and <50 mg/dL [<1.3 mmol/L] for women)*	2,012 (10.2)	1,245 (10.5)	767 (9.7)
Elevated blood pressure*			
*(systolic ≥130 and/or ≥85 diastolic mmHg or antihypertensive drug treatment)*	14,228 (72.3)	7,450 (63.1)	6,778 (85.9)
Elevated fasting glucose*			
*(≥110 mg/dL [≥6.1 mmol/L] or drug treatment for diabetes)*	6,761 (34.3)	3,371 (28.6)	3,390 (43.0)

Analysis was based on Mann–Whitney U test; Pearson Chi-square test. *P-values <0.05.

*PA, physical activity*.

### Sleep Duration and MetS

Results are summarized in Table 2. A significant interaction between sleep duration and age (in years) was observed (*P* = 0.009). Similar results were obtained for the interaction between sleep duration and age when the latter was treated as binary variable (*P* = 0.028). Compared to normal sleep duration, long sleep duration increased the prevalence of MetS in both middle-aged and old participants (*P* = 0.008 and *P* < 0.001, respectively). In contrast, short sleep duration was linked to a higher prevalence of MetS in middle-aged but not older subjects (*P* < 0.001 for middle-aged subjects; *P* = 0.716 for older subjects).

**Table 2:** Associations between sleep variables and metabolic syndrome (MetS) in the Swedish EpiHealth cohort study.

	MetS			MetS			MetS		
						
	Absent, *n*(%)	Present, *n*(%)	PR (95% CI)	Absent, *n*(%)	Present,*n*(%)	PR (95% CI)	Absent,*n*(%)	Present, *n*(%)	PR (95% CI)
**Sleep duration (h/day)**	**Total (***n*** = 19,691)**			**Age <65 years (***n*** = 11,804)**			**Age ≥65 years (***n*** = 7,887)**		

≤6	4,674 (31.7)	1,712 (34.6)	**1.08 (1.03–1.13)**	3,067 (32.9)	965 (38.9)	**1.13 (1.06–1.22)**	1,607 (29.6)	747 (30.3)	1.01 (0.94–1.09)
7–8	9,576 (64.9)	2,962 (59.9)	Ref	6,040 (64.8)	1,426 (57.5)	Ref	3,536 (65.2)	1,536 (62.4)	Ref
≥9	500 (3.4)	267 (5.4)	**1.26 (1.14–1.39)**	218 (2.3)	88 (3.5)	**1.26 (1.06–1.50)**	282 (5.2)	179 (7.3)	**1.26 (1.12–1.42)**

**Sleep disturbance^a^**	**Total (***n*** = 19,142)**			**Age <65 years (***n*** = 11,553)**			**Age ≥65 years (***n*** = 7,589)**		

No	11,182 (77.7)	3,518 (74.0)	Ref	7,076 (77.4)	1,757 (72.8)	Ref	4,106 (78.3)	1,761 (75.2)	Ref
Yes	3,205 (22.3)	1,237 (26.0)	**1.12 (1.06–1.18)**	2,065 (22.6)	655 (27.2)	**1.14 (1.05–1.23)**	1,140 (21.7)	582 (24.8)	**1.11 (1.03–1.20)**

**Sleep-disordered breathing symptoms^a^**	**Total (***n*** = 16,467)**			**Age <65 years (***n*** = 10,059)**			**Age ≥65 years (***n*** = 6,408)**		

No	10,583 (84.6)	2,870 (72.6)	Ref	6,866 (85.2)	1,419 (70.8)	Ref	3,717 (83.3)	1,451 (74.5)	Ref
Yes	1,933 (15.4)	1,081 (27.4)	**1.44 (1.36–1.53)**	1,189 (14.8)	585 (29.2)	**1.50 (1.38–1.63)**	744 (16.7)	496 (25.5)	**1.36 (1.26–1.47)**

*The results derived from log-binomial regression analysis. This analysis was controlled for participants’ exact age (in years), gender, educational level, physical activity during leisure time, smoking status, and alcohol consumption*.

*^a^Participants reported that at least one symptom (see methods for description) occurred ≥4 times per week. Bold values = *P*-values <0.05*.

*PR, prevalence ratio; Ref, reference group for the analysis*.

A secondary analysis was performed dividing the sleep duration variable into four instead of three categories (i.e., ≤5; 6; 7–8; and ≥9 h per day). In the entire sample, for those who reported to sleep ≤5 or ≥9 h per day a higher prevalence of MetS was found (PR [95% CI]; ≤5 h: 1.16 [1.07–1.25]; and ≥9 h: 1.26 [1.14–1.39]), compared with those who slept between 7 and 8 h per day. No such difference in the prevalence of MetS was observed between the 6 h-sleep duration and reference groups (1.05 [0.99–1.10]). When stratified by age, a similar pattern was observed among middle-aged subjects (≤5 h: 1.29 [1.17–1.43]; 6 h: 1.08 [0.99–1.16]; and ≥9h: 1.27 [95% CI 1.07–1.50]). In contrast, in the ≥65 years age group only long sleep duration was linked to a higher prevalence of MetS (1.26 [CI 1.12–1.42]).

### Sleep Disturbance and MetS

Results are summarized in Tables 2 and 3. No significant interaction between sleep disturbance and age was observed [*P* = 0.189 for sleep disturbance × age (in years); *P* = 0.521 for sleep disturbance × age group]. Participants with sleep disturbance were more likely to have MetS, than those without sleep disturbance (*P* < 0.001; Table 2). A separate analysis in the entire cohort (i.e., including both age groups) demonstrated that the number of sleep disturbance symptoms showed a positive association with the prevalence of MetS (Table 3).

**Table 3:** Association between the number of self-reported sleep disturbance symptoms and prevalence of MetS.

	MetS		
		
Number of sleep disturbance symptoms	Absent,*n*(%)	Present, *n*(%)	PR (95% CI)
0	11,182 (77.7)	3,518 (74.0)	Ref
1–2	2,530 (17.6)	919 (19.3)	**1.08 (1.02–1.15)**
3–4	675 (4.7)	318 (6.7)	**1.25 (1.14–1.37)**
1–2	2,530 (78.9)	919 (74.3)	Ref
3–4	675 (21.1)	318 (25.7)	**1.15 (1.04–1.28)**

*The results derived from log-binomial regression analysis. This analysis was controlled for participants’ exact age (in years), gender, educational level, physical activity during leisure time, smoking status, and alcohol consumption. Note that the analysis was performed in the entire group, as the interaction between age and sleep disturbance did not reach significance (*P* > 0.05). Bold values = *P*-values <0.05*.

*PR, prevalence ratio; Ref, reference group for the analysis; MetS, metabolic syndrome*.

### SDB Symptoms and MetS

Sleep-disordered breathing symptoms were associated with a higher prevalence of MetS in both age groups (*P* < 0.05; Table 2). However, the prevalence of MetS was higher among middle-aged subjects with SDB, as compared to older subjects with SDB (*P* < 0.001 for SDB × age). A separate analysis in the entire cohort, as well as within the age groups, revealed that the higher the number of SDB symptoms, the higher the prevalence of MetS (Table 4).

**Table 4:** Association between the number of self-reported SDB symptoms and prevalence of MetS.

	MetS			MetS			MetS		
						
Number of SDB symptoms	Absent, *n*(%)	Present, *n*(%)	PR (95% CI)	Absent, *n*(%)	Present, *n*(%)	PR (95% CI)	Absent,*n*(%)	Present, *n*(%)	PR (95% CI)
	**Total (***n*** = 16,467)**			**Age <65 years (***n*** = 10,059)**			**Age ≥65 years (***n*** = 6,408)**		

0	10,583 (84.6)	2,870 (72.6)	Ref	6,866 (85.2)	1,419 (70.8)	Ref	3,717 (83.3)	1,451 (74.5)	Ref
1	1,489 (11.9)	687 (17.4)	**1.30 (1.22–1.40)**	922 (11.4)	389 (19.4)	**1.39 (1.26–1.53)**	567 (12.7)	298 (15.3)	**1.19 (1.08–1.31)**
2	444 (3.5)	394 (10.0)	**1.76 (1.64–1.90)**	267 (3.3)	196 (9.8)	**1.78 (1.59–1.99)**	177 (4.0)	198 (10.2)	**1.74 (1.57–1.92)**

	**Total (***n*** = 3,014)**			**Age <65 years (***n*** = 1,774)**			**Age ≥65 years (***n*** = 1,240)**		

1	1,489 (77.0)	687 (63.6)	Ref	922 (77.5)	389 (66.5)	Ref	567 (76.2)	298 (60.1)	Ref
2	444 (23.0)	394 (36.4)	**1.41 (1.28–1.54)**	267 (22.5)	196 (33.5)	**1.33 (1.17–1.52)**	177 (23.8)	198 (39.9)	**1.48 (1.30–1.69)**

*The results derived from log-binomial regression analysis. This analysis was controlled for participants’ exact age (in years), gender, educational level, physical activity during leisure time, smoking status, and alcohol consumption. Note that the analysis was performed in the entire group, as well as for both age groups separately (*P* < 0.001 for SDB symptoms × age). Bold values = *P*-values <0.05*.

*PR, prevalence ratio; Ref, reference group for the analysis; SDB, sleep-disordered breathing; MetS, metabolic syndrome*.

### Sensitivity Analysis

In a sensitivity analysis, a multiple imputation approach was used to investigate whether exclusions because of missing values may have influenced our results. The associations between sleep parameters and the prevalence of MetS (revealed by our main analyses, see Table 2) remained significant, both in the entire sample and when stratified by age group (data not shown).

As mentioned above, significant interactions between age and sleep duration, as well as between age and SDB symptoms were found. To further examine the influence of age on observed associations between these sleep parameters and the prevalence of MetS, an additional sensitivity analysis dividing subjects into three instead of two age categories (early midlife: 45–54 years; older midlife: 55–64 years; and older ≥65 years old) was performed. This analysis demonstrated that early and older midlife age groups had a higher prevalence of MetS when reporting short sleep duration (≤6 h per day). In contrast, long sleep duration (≥9 h per day) was linked to a higher prevalence of MetS only in the older midlife age group and among individuals ≥65 years old (see Table S1 in Supplementary Material). Finally, in all age groups an association between SDB symptoms and increased prevalence of MetS was noticed, with highest PR for subjects of the early midlife age group (see Table S1 in Supplementary Material).

## Discussion

Our study demonstrates that the association between sleep duration and MetS varies by age. In middle-aged participants (45–65 years), both short (defined as ≤6 h sleep per day) and long (defined as ≥9 h sleep per day) duration sleepers exhibited an increased prevalence of MetS, compared with normal-duration sleepers (defined as 7–8 h sleep per day). In contrast, in older individuals (aged ≥65 years), long but not short sleep duration was linked to a higher likelihood of having MetS. Collectively, our study provides an important piece of evidence that the relation between sleep duration and metabolic health may change during adulthood. Age-specific associations between sleep duration and MetS components have also been observed by others. For instance, a study using the NHANES I follow-up data showed that daily sleep durations of ≤5 h compared to 7–8 h were associated with a significantly increased risk of incident hypertension in participants aged 32–59 years. This association was, however, not found among people aged ≥60 years (20). In a cross-sectional study of 29,333 individuals at age ≥50 years, it has further been shown that long sleep (≥9 h/days) but not short sleep was associated with an increased risk of MetS (21).

Sleep-disordered breathing comprises alterations in respiratory rate, rhythm, and depth present during sleep (22). Obstruction of the upper airway during sleep has been attributed to hypoxia, pulmonary hypertension, and light sleep (8, 23), all of which may cause metabolic perturbations (3, 8, 24–27). In this study, we show that both middle-aged and older subjects were at higher risk of MetS, when suffering from at least one SDB symptom ≥4 times per week. This association became stronger the higher the number of SDB symptoms. Additionally, our analysis revealed that middle-aged individuals with SDB exhibited a higher risk of MetS, than older subjects with SDB. One possible explanation for the latter finding could be that older humans, due to sleep hallmarked by lighter sleep stages and reduced time in rapid eye movement (REM) sleep (28), may run a lower risk to suffer from apneas during REM, than middle-aged subjects. Apneas during REM sleep have been proposed to be particularly detrimental to metabolic health (29–31). Overall, our findings could suggest that screening for SDB symptoms, e.g., by means of questionnaires or anamnestic interviews, may be particularly relevant for metabolic risk assessment in middle-aged people.

Another finding of our study was that reports of sleep disturbances, including difficulties in initiating and maintaining sleep, increased the prevalence of MetS. In contrast to sleep duration and SDB symptoms, associations between sleep disturbances and MetS did not differ between age groups. Relationships between measures of sleep disturbance and MetS have also been described by others (6, 9). For instance, data from a nationwide epidemiological survey conducted on middle-aged residents (mean age <60 years; *n* = 4,197) showed that problems with falling and staying asleep were associated with an increased prevalence of MetS (24 and 28%, respectively) (9). In a separate study involving 210 volunteers with a mean age of 46 years, it was shown that self-reported global sleep quality, measured with the Pittsburgh Sleep Quality Index, was related to MetS (6). Specifically, an increase of the global sleep score of 2.6 points was associated with an odds of 1.44 of having MetS (6). However, it must be noted that there are also negative results. In a study of 796 Taiwanese male police officers (mean age 37.4 years), no association between sleep quality and MetS was observed (32).

### Strengths and Limitations

A major strength of this study is that the analysis was based on a relatively large sample. Moreover, results were robust to adjustments for multiple potential confounders, such as lifestyle factors. To our best knowledge, our study is also among the first to investigate systematically how various characteristics of poor sleep link to the risk of MetS in middle-aged and older subjects. Several limitations, however, apply to our cross-sectional study. It cannot prove cause and effect. Another limitation is that sleep variables were based on self-reports. Thus, our observations should be confirmed by further studies utilizing objectively measured sleep parameters. An additional limitation of our study is that no measures of circadian misalignment have been collected. Sleeping 7–8 h during circadian improper time windows (e.g., during daytime because of night shift work) has been shown to adversely affect metabolic health (33, 34). Moreover, other potential confounders, such as pathological conditions (e.g., chronic or acute pain) and sleep-related medication, have not been included in the present analysis. It must also be noted that criteria underlying the definition of components of MetS (e.g., hypertension) can vary between studies. Finally, most of participants were of northern European origin which may reduce the generalizability of our results to other ethnic groups.

### Conclusion

In our study, we demonstrate that both sleep duration outside 7-8 hours per day and having at least one SDB symptom ≥4 times per week increase the risk of MetS in an age-specific manner. Sleep disturbances (i.e., at least one symptom ≥4 times per week) were also associated with an increased prevalence of MetS. The latter relationship was not modified by subject’s age. Given the high prevalence of sleep problems and metabolic perturbations in modern society, educational programs aiming to optimize sleep could, therefore, represent promising interventions to improve metabolic health in middle-aged and older subjects.

## Ethics Statement

The Ethics Committee at Uppsala University approved the general procedures of the EpiHealth study. All subjects gave written informed consent in accordance with the Declaration of Helsinki. An additional ethical approval for the current data analyses was obtained from the Ethics Committee at Uppsala University. A short description of the research proposal was displayed on EpiHealth’s homepage for 1 month which allowed participants in EpiHealth study to withdraw their consent.

## Author Contributions

OT: wrote the manuscript, performed the literature search, data and statistical analysis, and data interpretation. EL: reviewed and revised the manuscript, and supported data interpretation. SE: contributed to the study design, acquired data, and gave scientific advice. LL: contributed to the study design, acquired data, and reviewed the manuscript. HS: reviewed and revised the manuscript. CB: reviewed and revised the manuscript, supported data interpretation, and gave scientific advice. All authors approved the final version of the manuscript. OT and CB had full access to all of the data and take responsibility for the integrity and accuracy of the data analysis.

## Conflict of Interest Statement

The authors declare that the research was conducted in the absence of any commercial or financial relationships that could be construed as a potential conflict of interest.

## References

[1] Alberti KG Eckel RH Grundy SM Zimmet PZ Cleeman JI Donato KA Harmonizing the metabolic syndrome: a joint interim statement of the International Diabetes Federation task force on epidemiology and prevention; National Heart, Lung, and Blood Institute; American Heart Association; World Heart Federation; International Atherosclerosis Society; and international association for the study of obesity. Circulation (2009) 120(16):1640–5. 10.1161/CIRCULATIONAHA.109.192644 19805654

[2] Hall MH Muldoon MF Jennings JR Buysse DJ Flory JD Manuck SB. Self-reported sleep duration is associated with the metabolic syndrome in midlife adults. Sleep (2008) 31(5):635–43. 10.1093/sleep/31.5.635 18517034 PMC2398755

[3] Koren D Dumin M Gozal D. Role of sleep quality in the metabolic syndrome. Diabetes Metab Syndr Obes (2016) 9:281–310. 10.2147/DMSO.S95120 27601926 PMC5003523

[4] Song Q Liu X Zhou W Wang X Wu S. Changes in sleep duration and risk of metabolic syndrome: the Kailuan prospective study. Sci Rep (2016) 6:36861. 10.1038/srep36861 27857185 PMC5114677

[5] Ju SY Choi WS. Sleep duration and metabolic syndrome in adult populations: a meta-analysis of observational studies. Nutr Diabetes (2013) 3:e65. 10.1038/nutd.2013.8 23670223 PMC3671750

[6] Jennings JR Muldoon MF Hall M Buysse DJ Manuck SB. Self-reported sleep quality is associated with the metabolic syndrome. Sleep (2007) 30(2):219–23. 10.1093/sleep/30.2.219 17326548

[7] Theorell-Haglow J Berne C Janson C Lindberg E. The role of obstructive sleep apnea in metabolic syndrome: a population-based study in women. Sleep Med (2011) 12(4):329–34. 10.1016/j.sleep.2010.06.014 21345723

[8] Lam JC Mak JC Ip MS. Obesity, obstructive sleep apnoea and metabolic syndrome. Respirology (2012) 17(2):223–36. 10.1111/j.1440-1843.2011.02081.x 21992649

[9] Lin SC Sun CA You SL Hwang LC Liang CY Yang T The link of self-reported insomnia symptoms and sleep duration with metabolic syndrome: a Chinese population-based study. Sleep (2016) 39(6):1261–6. 10.5665/sleep.5848 27070137 PMC4863215

[10] Tseng PH Lee PL Hsu WC Ma Y Lee YC Chiu HM A higher proportion of metabolic syndrome in Chinese subjects with sleep-disordered breathing: a case-control study based on electrocardiogram-derived sleep analysis. PLoS One (2017) 12(1):e0169394. 10.1371/journal.pone.0169394 28081171 PMC5231382

[11] Qian Y Xu H Wang Y Yi H Guan J Yin S. Obstructive sleep apnea predicts risk of metabolic syndrome independently of obesity: a meta-analysis. Arch Med Sci (2016) 12(5):1077–87. 10.5114/aoms.2016.61914 27695500 PMC5016589

[12] Nakajima K Suwa K Toyama K. Age-dependent changes in the association between sleep duration and impaired glucose metabolism. World J Diabetes (2017) 8(8):397–406. 10.4239/wjd.v8.i8.397 28861177 PMC5561039

[13] Kim J Jo I. Age-dependent association between sleep duration and hypertension in the adult Korean population. Am J Hypertens (2010) 23(12):1286–91. 10.1038/ajh.2010.166 20706198

[14] Lopez-Garcia E Faubel R Guallar-Castillon P Leon-Munoz L Banegas JR Rodriguez-Artalejo F. Self-reported sleep duration and hypertension in older Spanish adults. J Am Geriatr Soc (2009) 57(4):663–8. 10.1111/j.1532-5415.2009.02177.x 19392958

[15] Titova OE Lindberg E Elmstahl S Lind L Schioth HB Benedict C. Association between shift work history and performance on the trail making test in middle-aged and elderly humans: the EpiHealth study. Neurobiol Aging (2016) 45:23–9. 10.1016/j.neurobiolaging.2016.05.007 27459922

[16] Lind L Elmstahl S Bergman E Englund M Lindberg E Michaelsson K EpiHealth: a large population-based cohort study for investigation of gene-lifestyle interactions in the pathogenesis of common diseases. Eur J Epidemiol (2013) 28(2):189–97. 10.1007/s10654-013-9787-x 23435790

[17] Alberti KG Zimmet PZ. Definition, diagnosis and classification of diabetes mellitus and its complications. Part 1: diagnosis and classification of diabetes mellitus provisional report of a WHO consultation. Diabet Med (1998) 15(7):539–53. 10.1002/(SICI)1096-9136(199807)15:7<539::AID-DIA668>3.0.CO;2-S 9686693

[18] Barros AJ Hirakata VN. Alternatives for logistic regression in cross-sectional studies: an empirical comparison of models that directly estimate the prevalence ratio. BMC Med Res Methodol (2003) 3:21. 10.1186/1471-2288-3-21 14567763 PMC521200

[19] Textor J Hardt J Knuppel S DAGitty: a graphical tool for analyzing causal diagrams. Epidemiology (2011) 22(5):745 10.1097/EDE.0b013e318225c2be 21811114

[20] Gangwisch JE Heymsfield SB Boden-Albala B Buijs RM Kreier F Pickering TG Short sleep duration as a risk factor for hypertension: analyses of the first National Health and Nutrition Examination Survey. Hypertension (2006) 47(5):833–9. 10.1161/01.HYP.0000217362.34748.e0 16585410

[21] Arora T Jiang CQ Thomas GN Lam KB Zhang WS Cheng KK Self-reported long total sleep duration is associated with metabolic syndrome: the Guangzhou Biobank Cohort study. Diabetes Care (2011) 34(10):2317–9. 10.2337/dc11-0647 21873559 PMC3177714

[22] Baekey DM Feng P Decker MJ Strohl KP. Breathing and sleep: measurement methods, genetic influences, and developmental impacts. ILAR J (2009) 50(3):248–61. 10.1093/ilar.50.3.248 19506312

[23] Young T Peppard PE Taheri S Excess weight and sleep-disordered breathing. J Appl Physiol (1985) (2005) 99(4):1592–9. 10.1152/japplphysiol.00587.2005 16160020

[24] Barcelo A Miralles C Barbe F Vila M Pons S Agusti AG. Abnormal lipid peroxidation in patients with sleep apnoea. Eur Respir J (2000) 16(4):644–7. 10.1034/j.1399-3003.2000.16d13.x 11106206

[25] Dyugovskaya L Lavie P Lavie L. Increased adhesion molecules expression and production of reactive oxygen species in leukocytes of sleep apnea patients. Am J Respir Crit Care Med (2002) 165(7):934–9. 10.1164/ajrccm.165.7.2104126 11934717

[26] Lavie L Oxidative stress – a unifying paradigm in obstructive sleep apnea and comorbidities. Prog Cardiovasc Dis (2009) 51(4):303–12. 10.1016/j.pcad.2008.08.003 19110132

[27] Tan X van Egmond L Chapman CD Cedernaes J Benedict C Aiding sleep in type 2 diabetes: therapeutic considerations. Lancet Diabetes Endocrinol (2017) 6(1):60–8. 10.1016/S2213-8587(17)30233-4 28844889

[28] Ohayon MM Carskadon MA Guilleminault C Vitiello MV. Meta-analysis of quantitative sleep parameters from childhood to old age in healthy individuals: developing normative sleep values across the human lifespan. Sleep (2004) 27(7):1255–73. 10.1093/sleep/27.7.1255 15586779

[29] Mokhlesi B Ayas NT Cardiovascular events in obstructive sleep apnea – can CPAP therapy SAVE lives? N Engl J Med (2016) 375(10):994–6. 10.1056/NEJMe1609704 27571490

[30] Mokhlesi B Varga AW Obstructive sleep apnea and cardiovascular disease: REM sleep matters! Am J Respir Crit Care Med (2017) 197(5):554–6. 10.1164/rccm.201710-2147ED PMC6005246 29141154

[31] Reutrakul S Mokhlesi B. Obstructive sleep apnea and diabetes: a state of the art review. Chest (2017) 152(5):1070–86. 10.1016/j.chest.2017.05.009 28527878 PMC5812754

[32] Chang JH Huang PT Lin YK Lin CE Lin CM Shieh YH Association between sleep duration and sleep quality, and metabolic syndrome in Taiwanese police officers. Int J Occup Med Environ Health (2015) 28(6):1011–23. 10.13075/ijomeh.1896.00359 26294202

[33] Cedernaes J Schioth HB Benedict C. Determinants of shortened, disrupted, and mistimed sleep and associated metabolic health consequences in healthy humans. Diabetes (2015) 64(4):1073–80. 10.2337/db14-1475 25805757

[34] Qian J Scheer FA Circadian system and glucose metabolism: implications for physiology and disease. Trends Endocrinol Metab (2016) 27(5):282–93. 10.1016/j.tem.2016.03.005 27079518 PMC4842150

