# Supplementary material for: Associations Between the Prevalence of Metabolic Syndrome and Sleep Parameters Vary by Age
Source: Front Endocrinol (Lausanne). 2018 May 11;9:234. doi: 10.3389/fendo.2018.00234 (PMC5958301; doi:10.3389/fendo.2018.00234)
Supplement: Supplementary file 1 [file table_1.PDF]

Supplementary Table 1: Associations between sleep duration, SDB symptoms and metabolic syndrome in the Swedish EpiHealth cohort study, stratified by age

	Metabolic syndrome			Metabolic syndrome			Metabolic syndrome		
	Absent, n (%)	Present, n (%)	PR (95% CI)*	Absent, n (%)	Present, n (%)	PR (95% CI)*	Absent, n (%)	Present, n (%)	PR (95% CI)*
Sleep duration (h/day)	Age 45-54 y (n= 5,552)			Age 55-64 y (n=6,252)			Age ≥ 65 y (n=7,887)		
≤ 6	1,488 (32.0)	365 (40.6)	**1.17 (1.04-1.32)**	1,579 (33.8)	600 (38.0)	**1.10 (1.01-1.20)**	1,607 (29.6)	747 (30.3)	1.01 (0.94-1.09)
7-8	3,068 (65.9)	507 (56.4)	Ref	2,972 (63.6)	919 (58.2)	Ref	3,536 (65.2)	1,536 (62.4)	Ref
≥9	97 (2.1)	27 (3.0)	1.34 (0.97-1.86)	121 (2.6)	61 (3.9)	**1.26 (1.03-1.55)**	282 (5.2)	179 (7.3)	**1.26 (1.12-1.42)**
SDB symptoms #	Age 45-54 y (n= 4,830)			Age 55-64 y (n=5,229)			Age ≥ 65 y (n=6,408)		
No	3,613 (88.2)	534 (73.0)	Ref	3,253 (82.2)	885 (69.6)	Ref	3,717 (83.3)	1,451 (74.5)	Ref
Yes	485 (11.8)	198 (27.0)	**1.54 (1.33-1.78)**	704 (17.8)	387 (30.4)	**1.47 (1.33-1.62)**	744 (16.7)	496 (25.5)	**1.36 (1.26-1.47)**

The results derived from log-binomial regression analysis. This analysis was controlled for participants' exact age (in years), gender, educational level, physical activity during leisure time, smoking status, and alcohol consumption. # Participants reported that at least one symptom (see methods for description) occurred ≥4 times per week. Bold values = P-values < 0.05. *Abbreviations:* PR, prevalence ratio; Ref, reference group for the analysis.
